# ‘Normalizing' the malignant phenotype of luminal breast cancer cells via alpha(v)beta(3)-integrin

**DOI:** 10.1038/cddis.2016.387

**Published:** 2016-12-01

**Authors:** Hanan Abu-Tayeh, Keren Weidenfeld, Alisa Zhilin-Roth, Sagi Schif-Zuck, Sonja Thaler, Cristina Cotarelo, Tuan Z Tan, Jean P Thiery, Jeffrey E Green, Geula Klorin, Edmond Sabo, Jonathan P Sleeman, Maty Tzukerman, Dalit Barkan

**Affiliations:** 1 Department of Human Biology, University of Haifa, Haifa, Israel; 2 Medical Faculty Mannheim, Centre for Biomedicine and Medical Technology Mannheim (CBTM), University of Heidelberg, Mannheim, Germany; 3 Department of Pathology, University Medical Center Mainz, Langenbeckstr, Mainz, Germany; 4 Cancer Science Institute of Singapore, National University of Singapore, Singapore, Singapore; 5 Department of Biochemistry, Yong Loo Lin School of Medicine, National University of Singapore, Singapore, Singapore; 6 Institute of Molecular and Cell Biology, A*STAR, Singapore, Singapore; 7 Laboratory of Cancer Biology and Genetics, National Cancer Institute, Bethesda, MD, USA; 8 Department of Pathology, Rambam Medical Center, Haifa, Israel; 9 Karlsruhe Institute of Technology (KIT), Campus Nord, Institut für Toxikologie und Genetik, Karlsruhe, Germany; 10 Rappaport Faculty of Medicine and Research Institute, Technion – Israel Institute of Technology, Haifa, Israel

## Abstract

Reestablishing tissue organization of breast cancer cells into acini was previously shown to override their malignant phenotype. In our study, we demonstrate that alpha(v)beta(3) integrin (Int-*α*v*β*3), previously shown to play a role in cancer progression, promoted differentiation and growth arrest of organoids derived from luminal A breast cancer cells grown in their relevant three-dimensional microenvironment. These organoids differentiated into normal-like acini resembling a benign stage of breast tissue. Likewise, we demonstrate that Int-*α*v*β*3 is selectively expressed in the epithelium of the benign stage of breast tissues, and is lost during the early stages of luminal A breast cancer progression. Notably, the organoids' reversion into normal-like acini was mediated by cancer luminal progenitor-like cells expressing both EpCAM^high^CD49f^low^CD24^+^ and Int-*α*v*β*3. Furthermore, downregulation of Notch4 expression and downstream signaling was shown to mediate Int-*α*v*β*3-induced reversion. Intriguingly, when luminal A breast cancer cells expressing Int-*α*v*β*3 were injected into a humanized mouse model, differentiated tumors developed when compared with that generated by control cells. Hence, our data suggest that promoting differentiation of luminal A breast cancer cells by signaling emanating from Int-*α*v*β*3 can potentially promote ‘normalization' of their malignant phenotype and may prevent the malignant cells from progressing.

Integrins are a large family of *αβ* heterodimeric cell surface receptors that mediate cell–cell and cell–extracellular matrix interactions^1, 2^ and play an important role during cancer progression. Of these receptors, *α*v*β*3 integrin (Int-*α*v*β*3) was shown to be highly expressed in several cancer types.^3, 4^ However, its contribution to breast cancer progression has been un-conclusive given conflicting results in the literature concerning the outcome of inhibiting Int-*α*v*β*3 expression/activity on breast tumor cells.^5, 6^ Recently, low concentrations of RGD-mimetic Int-*α*v*β*3 and integrin-*α*v*β*5 (Int-*α*v*β*5) inhibitors were shown to paradoxically stimulate tumor growth and angiogenesis.^5, 7, 8^ Moreover, regulation of tumor progression by tumor and stromal *β*3 integrin (Int*-β*3) were shown to vary in breast cancer models.^9^ Here we demonstrate that Int-*α*v*β*3 is selectively expressed in the epithelium of the benign stage of breast tissues and is lost during early stages of luminal A (positive for estrogen and progesterone receptors) breast cancer progression. Hence, these surprising results suggest that Int*-β*3 expression might maintain the differentiated state of premalignant tissue via its engagement with its restrictive normal microenvironment, the latter acting as a gatekeeper during neoplastic progression.^10^ Therefore, we hypothesized that Int-*β*3 re-expression in luminal A breast cancer cells will promote their differentiation in conjunction with their microenvironment.

We found that re-expression of Int*-β*3 by human luminal breast cancer cell lines MCF-7 and T47D promotes their cancer luminal progenitor-like cells (CLPs) to revert into growth-arrested acinar-like organoids resembling normal breast tissue when cultured in a three-dimensional (3D) reconstituted basement membrane extract (BME)^11^ mimicking the components of the normal basement membrane.^12^ This reversion was mediated by downregulation of Notch4 expression and its downstream signaling. Intriguingly, tumors developed by MCF-7-Int*β*3 cell were highly differentiated compared with MCF-7 tumors, grown in an *in vivo* humanized mouse model.^13, 14^ All together, these findings demonstrate for the first time to our knowledge that CLPs, which are present in luminal A breast cancer cell lines and tissues,^15^ can be induced to differentiate into acinar-like organoids via expression of Int-*α*v*β*3 in the 3D system and *in vivo* to a more differentiated phenotype.

## Results

### Int-*β*3 is expressed in early hyperplastic breast tissue and can promote differentiation of luminal A breast cancer cells in the 3D BME system

**Figure 1:**
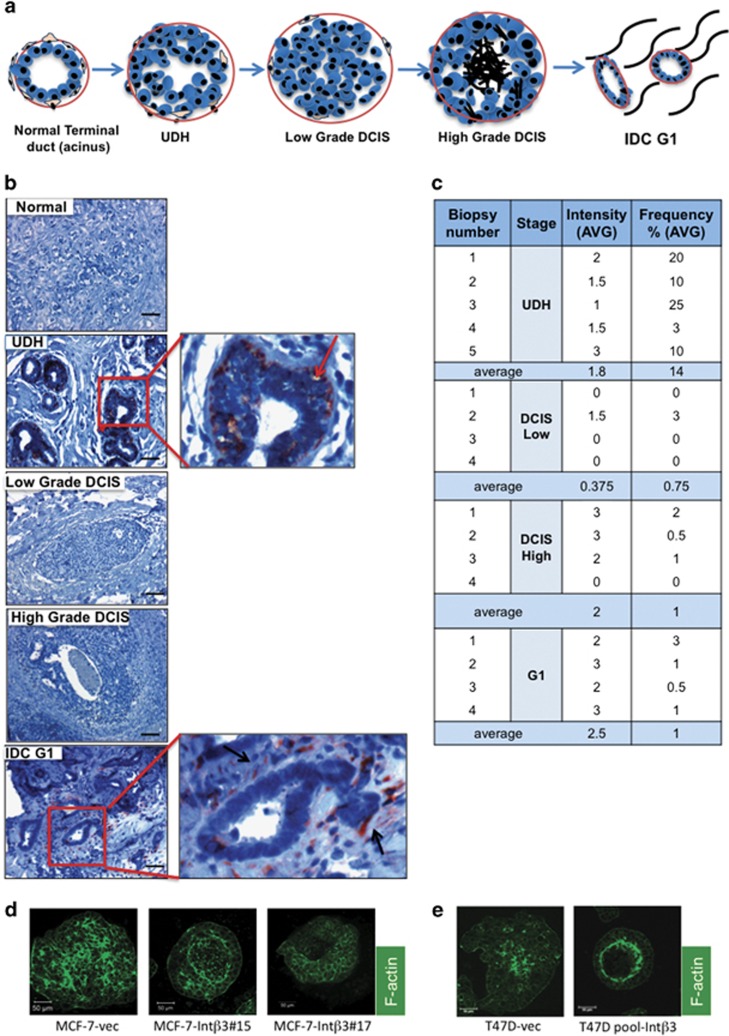
Int*-β*3 is expressed in UDH of human breast tissue and its expression induces a 3D-differentiated phenotype on luminal breast cancer cells *in vitro*. (**a**) Scheme demonstrating the different stages in breast cancer progression. (**b**) Paraffin-fixed tissue from normal and luminal A breast cancer patients representing different stages (4–5 cases of each stage) stained for Int-*β*3 expression (red) nuclei countered stained with hematoxilin (blue). Bars=100 *μ*m. (**c**) Intensity of staining and percentage of breast epithelial cells expressing Int-*β*3 (frequency). (**d**–**e**) F-actin staining (green) of MCF-7 (**d**) and T47D cell lines (**e**) cultured for 40 days in the 3D BME system. Representative confocal images, magnification × 40, bars= 50 *μ*m

We examined the expression of Int-*β*3 in paraffin-embedded biopsy tissues of normal breast and luminal A; usual ductal hyperplasia (UDH), low-grade ductal carcimoma in situ (DCIS), high-grade DCIS and invasive ductal carcinoma grade 1 (IDC-G1) (Figures 1a and b). Intriguingly, all UDH cases (14% of the luminal epithelial cell/acinus) expressed Int-*β*3, which was confined to the outer layer of the luminal epithelial cells comprising the acinus (Figures 1b and c and Supplementary Figure S1A, see red arrows). In contrast, Int-*β*3 expression was not detected in normal breast tissue and rarely in low, high-grade epithelium of DCIS and IDC-G1 samples (Figures 1b and c). However, moderate to high expression of Int-*β*3 was detected in the stroma of IDC-G1 samples (Figure 1b and Supplementary Figure S1B, see black arrows). Thus, Int*-β*3 expression in the breast epithelial cells was lost on progression from benign to IDC-G1 stage. Hence, Int-*β*3 expression may mediate engagement of the benign tissue with its restrictive normal microenvironment, the latter being a gatekeeper during neoplastic progression.^10^ Therefore, we tested whether re-expression of Int-*β*3 in MCF-7 and T47D luminal A breast cancer cell lines (Supplementary Figure S2) will promote their differentiation. Int-*β*3 stably expressed in clones of MCF-7 cells (MCF-7-Int*β*3 #15 and #17) and pool of T47D cell line were cultured in the 3D BME system. Intriguingly, both cell lines stably expressing Int-*β*3 differentiated into an acinar-like structure, similar to normal mammary tissue, whereas control MCF-7-vec and T47D-vec cells formed disorganized organoids (Figures 1d and e).

### Luminal breast cancer cells expressing Int-*α*v*β* are enriched with putative cancer luminal progenitor-like cells

Normal acini consist of luminal epithelial cells surrounding the lumen and myoepithelial cells surrounding the luminal cells. Both originate from mammary stem cells and progenitor cells. Three distinct types of human breast epithelial cell progenitors have been previously described: luminal-restricted, myoepithelial-restricted and bipotent progenitors.^16, 17, 18^ Int-*β*3 was recently found to serve as a cell surface marker specifically identifying the mouse luminal progenitors committed to terminally differentiate into the luminal epithelial cells.^19, 20^

**Figure 2:**
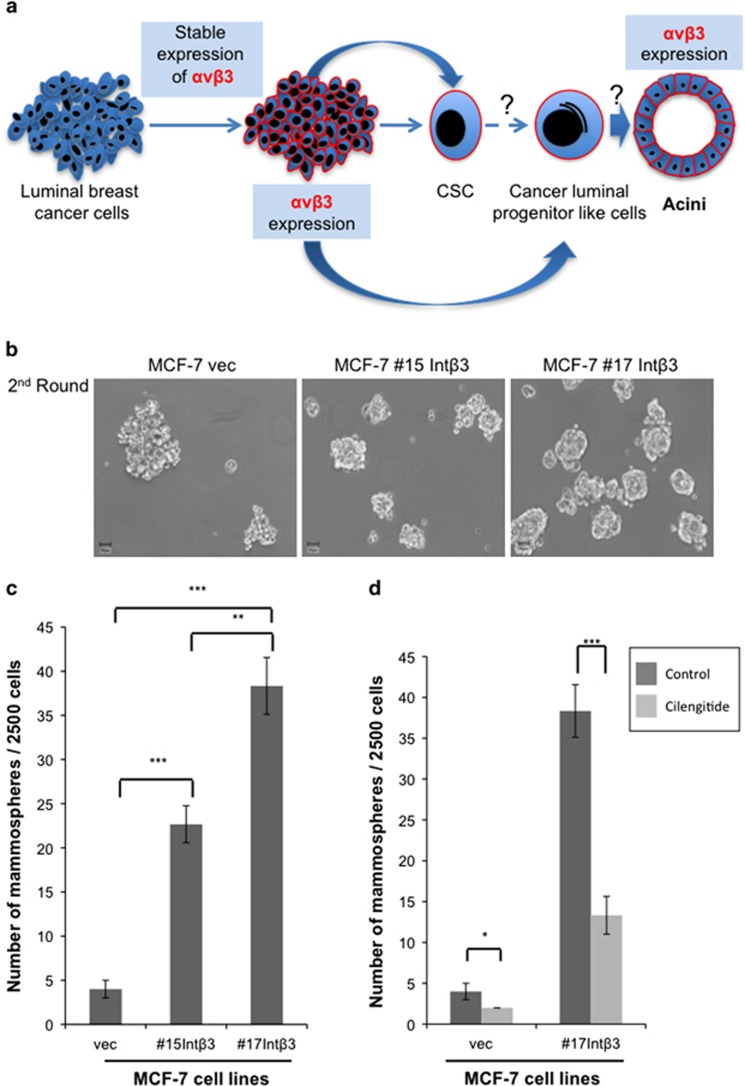
Characterizing MCF-7 cell lines for their sphere rising potential. (**a**) Scheme demonstrating the hypothesis: expression of Int-*α*v*β*3 in either CSC/CLPs will promote their differentiation into normal acini in the 3D BME system. (**b**–**d**) Second generation of grown mammospheres from MCF-7 cell lines. (**b**) Light microscopy images (magnification × 10). (**c**) Quantification of the number of mammospheres. (**d**) Quantification of mammospheres either untreated or treated with cilengitide (20 *μ*M). Columns; mean, bars; STD; *n*=3; *P⩽0.05; **P⩽0.01 and ***P⩽0.001

**Figure 3:**
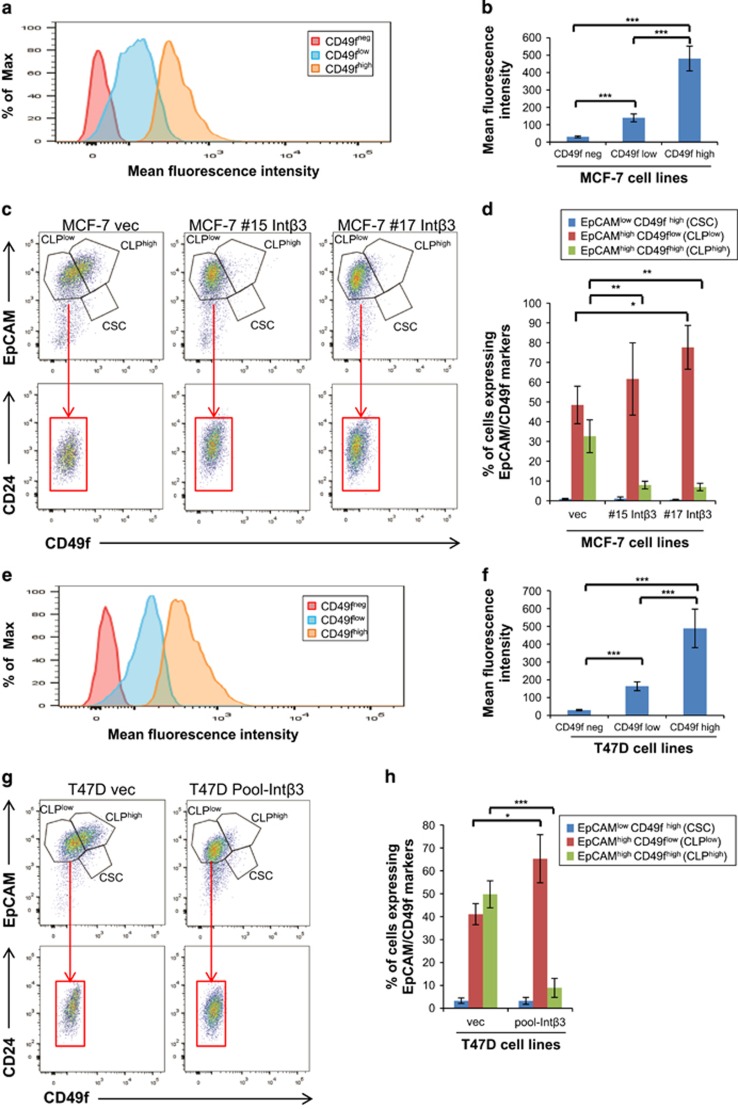
Int-*α*v*β*3 expression in MCF-7 and T47D cells promotes the enrichment of EpCAM^high^CD49f^low^CD24^+^ subpopulation of cells. Cells derived from second generation of grown mammopsheres of either (**a**–**d**) MCF-7 cell lines, or (**e**–**h**) T47D cell lines. (**a** and **e**) Histogram representing three cell populations with different levels of expression of CD49f normalized to unstained cells. (**b** and **f**) Mean fluorescence intensity of CD49f expression in the different subpopulations. (**c** and **g**) Top panel: representative dot plot showing CSC expressing EpCAM^low^CD49f^high^, CLPs either expressing EpCAM^high^CD49f^low^ (CLP^low^) or EpCAM^high^CD49f^high^ (CLP^high^) phenotype. Bottom panel (red square): representative dot plot showing CLP^low^ subpopulation positive for CD24 expression. (**d** and **h**) Percentage of CSC, CLP^low^ and CLP^high^. Quantification was carried out with FACSdiva software. Columns; mean, bars; STD; *n*=3; *P⩽0.05, **P⩽0.01 ***P⩽0.001

Similar to primary breast tumors and breast cancer cell lines, the MCF-7 and T47D cells consist of heterogeneous cell populations, including cancer stem cells (CSC) and cancer cells expressing markers of CLPs.^15^ Given previous studies and our surprising results demonstrating that Int-*β*3 can promote the differentiation of MCF-7 and T47D cell lines into a normal-like acini in the 3D BME system, has prompted us to test whether MCF-7-Int*β*3 cells may be enriched with cancer stem/progenitor-like cells that can commit and differentiate into normal-like acini (Figure 2a). Therefore, MCF-7 cell lines were grown as non-adherent mammospheres for two passages. MCF-7-Int*β*3 cell lines exhibited a significant increase in their sphere capacity formation (6–10-fold increase *versus* MCF-vec cells, Figures 2b and c), indicating their potential cancer stem/progenitor cell-like properties. Furthermore, cilengtide inhibited the sphere formation potential of both MCF-7-vec cells and, to a greater degree, MCF-7-Int*β*3 cells (Figure 2d). Given that cilengitide blocks Int-*α*v*β*3 and Int-*α*v*β*5 activity and that Int-*α*v*β*5 is expressed in both cell lines (data not shown), suggests that the sphere formation capacity of MCF-7-Int*β*3 cells is mediated partially by Int-*α*v*β*3. Next, we determined by flow cytometry analysis the distribution of cancer stem/progenitor-like cells in both the control and Int-*α*v*β*3 expressing MCF-7 and T47D cell lines. Single cells were dissociated from either 2D culture or secondary grown mammospheres (enriching for stem/progenitor-like cells) and were analyzed for the expression of (1) EpCAM^neg/low^ CD49f^high^ and CD44^high^ CD24^low^ phenotype; shown to be expressed by stem cells of normal and cancerous human breast tissue and breast cancer cell lines;^15, 21, 22, 23^ and for (2) EpCAM^pos^CD49f^pos^ phenotype; shown to be expressed by human luminal progenitor cells^24^ and to be present in MCF-7 and enriched in T47D cell lines.^15^ Our results demonstrate the presence of three subpopulations of cells across the cell lines; CSC and two subpopulations of cells with EpCAM^pos^CD49f^pos^ phenotype: EpCAM^high^CD49f^low^ (CLPs^low^) and EpCAM^high^CD49f^high^ (CLPs^high^) phenotype derived either from mammospheres (Figures 3a–c and e–g) or from 2D cultures (Supplementary Figures S3A–C and E–G). The CLPs^low^ were the prominent subpopulation across the different cell lines (Figures 3c, d, g and h; Supplementary Figures S3C–D and G–H). In addition, significant increase in CLPs^low^ and significant decrease in CLPs^high^ cells was apparent in both MCF-7-Int*β*3 #17 and T47D-Int*β*3 cells (Figures 3c and d and Figures 3g and h, respectively; Supplementary Figure S3G-H). Furthermore, CLPs^low^ expressing cells were all positive for CD24 expression (a luminal progenitor marker^24^) across all cell lines derived from mammospheres (Figures 3c and g) or from 2D culture (Supplementary Figures S3C and G), further confirming their potential CLPs phenotype. Importantly, within the EpCAM^high^CD49f^low^CD24^+^Int-*β*3^+^ cells derived from grown mammospheres of MCF-7 and T47D cell lines; 91–95% and 64% of the cells were positive for Int-*α*V*β*3 expression, respectively. Notably, the percentage of CSCs expressing either the EpCAM^neg/low^ CD49f^high^ (Figures 3d and h and Supplementary Figures S3D and H) or CD44^high^ CD24^low^ phenotype (Supplementary Figure S4) among the different cell lines did not increase and represented a small subpopulation of the total cell population (<10%). Hence, both cell lines non-expressing and expressing Int-αV*β*3 were predominantly enriched with CLPs^low^. However, in cells expressing Int-αV*β*3 and grown as mammospheres there was a significant increase in CLPs^low^.

### Int-av*β*3 expression in CLPs^low^ promotes their differentiation into acinar-like organoids in the 3D BME system

**Figure 4:**
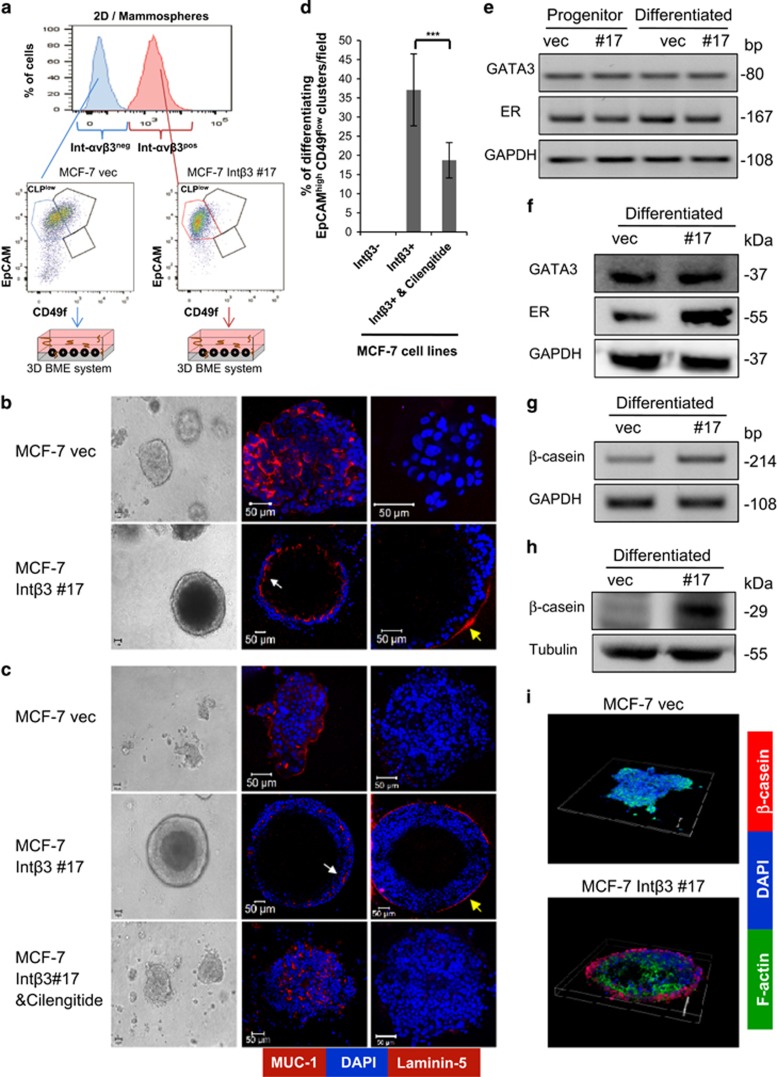
Int-αv*β*3 expression and activity is required to commit CLPs^low^ to differentiate into alveolar lineage in the 3D BME system. (**a**) Scheme demonstrating the sorting from 2D culture/mammospheres of CLP-Int-αv*β*3^neg^ or CLP-Int-*α*v*β*3^pos^ cells and their culture in the 3D BME system. (**b**) CLP-Int-*α*v*β*3^pos/neg^ derived from 2D culture or (**c**) derived from second generation of grown mammospheres either untreated or treated with cilengitide (40 *μ*M) and cultured in the 3D BME system. Left panel: representative light microscopy images (magnification × 20). Middle and right panel: representative confocal images of cross sections through the middle of organoids stained for MUC-1 (white arrow), laminin 5 (yellow arrow) and nuclei (Dapi, blue). Magnification × 40 (zooming was adjusted accordingly to attain the whole cluster in the field of view), bar=50 *μ*m, *n*=5. (**d**) Percentage of differentiated organoids per field in the 3D BME culture (an average of 10 fields were scored) ***P⩽0.001. (**e**) Semi-qPCR analysis of mRNA transcript levels of GATA3 and ER in sorted CLP-Int-*α*v*β*3^pos/neg^ before (progenitor) and after culturing in the 3D BME system (differentiated). (**f**) W.B. analysis for the expression of GATA3 and ER in CLP-Int-*α*v*β*3^pos/neg^ cultured in the 3D BME system. (**g**) Semi-qPCR analysis of mRNA transcript levels of *β*-casein in CLP-Int-*α*v*β*3^pos/neg^ cultured in the 3D BME system. (**h**) W.B. analysis for the expression of *β*-casein in CLP-Int-*α*v*β*3^pos/neg^ cultured in the 3D BME system (*n*=2). (**i**) Representative confocal images of z-stacks of organoids stained with Dapi for nuclei (blue) for F-actin (green) and immunofluorescence staining for *β*-casein (red). Magnification × 40. Bars=50 *μ*m. Representative results; *n*=3

**Figure 5:**
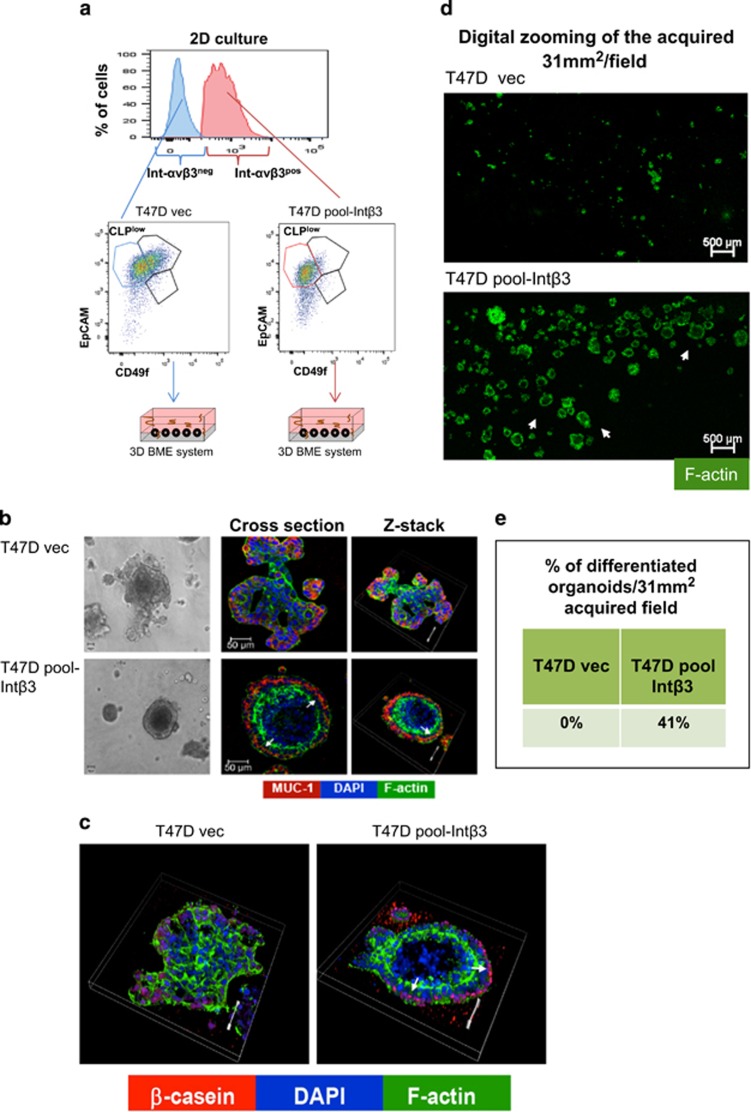
CLP-Int-*α*v*β*3^pos^ derived from T47D cells differentiate into acinar-like organoids expressing *β*-casein in the 3D BME system. (**a**–**e**) CLPs^low^ were derived from 2D culture. (**a**) Scheme demonstrating the sorting from 2D culture of either CLP-Int-αv*β*3^neg^ or CLP-Int-*α*v*β*3^pos^ cells and their culture in the 3D BME system. (**b**) Representative light microscopy images (left panel, magnification × 20) and confocal images (magnification × 40) of either cross sections through the middle of organoids (middle panel) or Z-stack images of the cells (right panel) cultured in the 3D BME system. Immunofluorescence staining for MUC-1 (red, see white arrow), Dapi for nuclei (blue) and F-actin (green) are presented. Bar=50 *μ*m. (**c**) Representative confocal images of z-stacks of organoids stained with Dapi for nuclei (blue), F-actin (green) and immunofluorescent staining for *β*-casein (red, see white arrow). Magnification × 40. Bars=50 *μ*m. Representative results; *n*=3. (**d**) Digital zooming of an acquired confocal images (31 mm^2^/field) stained with F-actin (green). White arrowheads indicate differentiated organoids. Magnification × 10. Bars=50 *μ*m. (**e**) Table representing percentage of differentiated organoids/31 mm^2^ acquired field

Given that non-expressing and expressing Int-αv*β*3 cells were enriched with CLPs^**low**^, but only cell lines expressing Int-αv*β*3 differentiated in the 3D BME system, has led us to test whether Int-αv*β*3 expression commits CLPs^**low**^ to differentiate into acinar-like organoids. CLPs^**low**^ subset either negative for Int-*α*V*β*3 expression (CLP-Int-αv*β*3^neg^; derived from MCF-7-vec and T47D-vec cell lines) or positive for Int-αV*β*3 expression (CLP-Int-αV*β*3^pos^; derived from MCF-7-Int*β*3 #15/#17 and T47D-Int*β*3 cell lines) were flow sorted either from 2D culture or from non-adherent mammospheres and were placed in the 3D BME system and scored for their potential to differentiate (Figures 4a and 5a). CLP-Int-*α*v*β*3^pos^ developed homogeneous spherical luminar-containing structures (Figures 4b and c, Figures 5b and d and Supplementary Figure S5) with apical expression of MUC-1 by both cell lines (Figures 4b and c middle panel, Figure 5b, white arrow) and basal expression of laminin 5 by MCF-7-Int*β*3 cells (Figures 3b and c right panel, yellow arrow.) Notably, 35% and 41% of CLP-Int-*α*v*β*3^pos^ derived either from MCF-7-Int*β*3 or T47D-Int*β*3 cells, respectively, differentiated (Figure 4d and Figures 5d and e). Whereas, CLP-Int-*α*v*β*3^neg^ formed disorganized cellular clusters (Figures 4b and c, Figure 5b–d and Supplementary Figure S5). Furthermore, treatment of CLP-Int-αv*β*3^pos^ with cilengitide significantly inhibited their differentiation into acinar-like structures (twofold reduction) compared with that of vehicle treated (Figures 4c and d). Since these acinar-like structures are derived from CLPs that were implicated previously as the source of basal-like breast tumors (negative for estrogen progesterone and Her2 receptor^24^), we investigated whether these Int-αv*β*3^pos^ acini retained their luminal phenotype. Indeed, flow sorted CLP-Int-αV*β*3^pos^ isolated from either non-adherent mammospheres (progenitors) or from 2D culture and cultured in the 3D BME system (differentiated) (Figures 4e and f) expressed the luminal markers GATA3 and estrogen receptor (ER). Intriguingly, the polarized outer layer of cells comprising the acini selectively expressed the milk protein *β*-casein in absence of a lactogenic stimulus (Figures 4g–i and 5c). In contrast, such a pattern of expression and localization were absent in the cellular organoids derived from CLP-Int-*α*v*β*3^neg^ (Figures 4i and 5c). These results suggest that expression of Int-αv*β*3 in CLPs^low^ derived either from 2D culture or from mammospheres can be redirected to differentiate into acinar-like structure reminiscent of the normal alveolar cells comprising the breast tissue.

### CLP-Int-av*β*3^pos^ differentiate into growth-arrested acinar-like organoids resembling UDH

**Figure 6:**
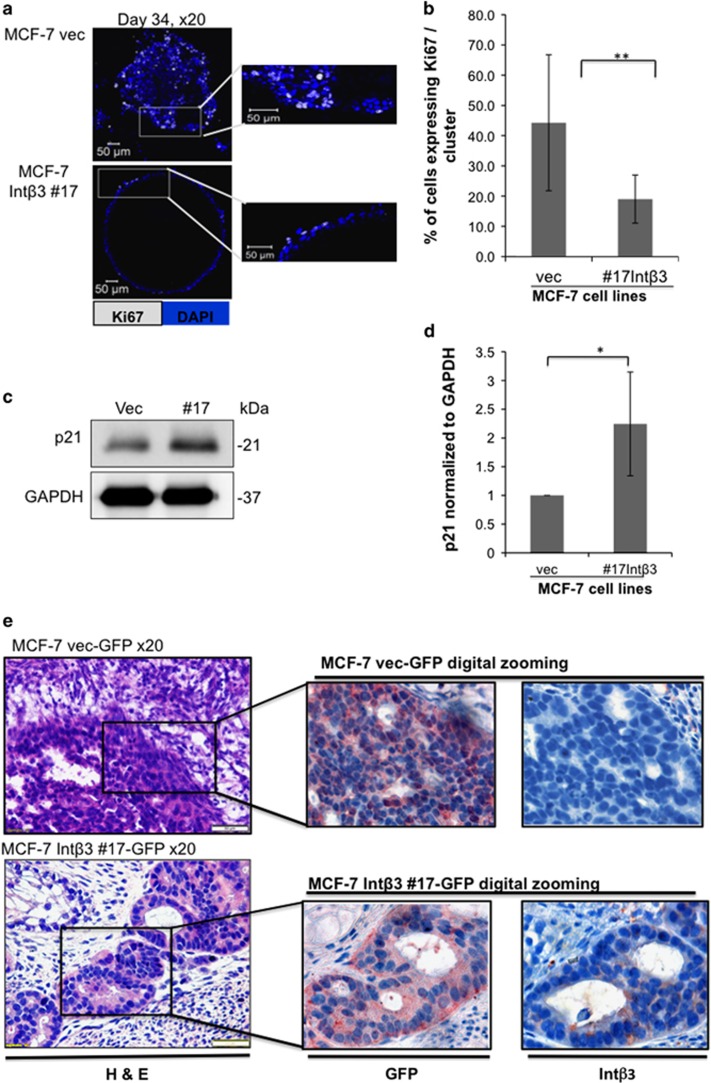
MCF-7-Int*β*3 cells are growth arrested in the 3D BME system and differentiate *in vivo*. (**a**–**d**) MCF-7 cell lines cultured in the 3D BME system. (**a**) Left panel: representative confocal image of cross section through the middle of an organoid (day 36) stained for Ki67 (white). Right panel: digital zooming of the selected area, white arrow indicates Ki67 positive cells. (**b**) Quantification of the percentage of Ki67-positive cells within each cross section through the middle of the organoids. Twenty-five organoids of each condition were scored. Bars=50 *μ*m. (**c**) W.B. analysis for the expression of p21 and its quantification normalized to GAPDH in the organoids (**d**); (*n*=3) (**e**) Serial paraffin section of teratomas injected with either MCF-7-vec-GFP or MCF-7-Int*β*3-GFP cells. Paraffin section were subjected to H&E staining (magnification × 20), or subjected to either GFP or Int*-β*3 staining (digital zooming of the selected area is presented). Representative images; *n*=3

During normal morphogenesis, cell clearing in the luminal space is mediated by cell death partly promoted by apoptosis.^25, 26, 27^ Indeed, at day 19 in culture we observed cell death in the center of the organoids (Supplementary Figure S6A-B), partly mediated by apoptosis (Supplementary Figure S6C). Furthermore, these acini-like structures were cell cycle arrested, depicted by significant decrease in the percentage of proliferating cells within each acini (Figures 6a and b) and significant increase in cyclin-dependent kinase inhibitor p21 expression (Figures 6c and d).

Importantly, nuclear size has been previously shown to increase from benign to malignant breast tissue along with disruption of the rounded architecture of the acini.^28^ Our results demonstrate a significant decrease in average nuclear size (*P*⩽0.01) comprising the differentiated acini, compared with undifferentiated organoids (Supplementary Figure S7A, lower panel). Furthermore, the average roundness values of both differentiated acini (derived from either normal breast MCF-10A cells or MCF-7-Int*β*3 #17 cells) were similar and significantly different from the average roundness value of the non-differentiated organoids (derived from MCF-7-vec cells) (Supplementary Figure S7B). Overall these results suggest that MCF-7-Int*β*3 #17 cells differentiated to a more benign stage resembling early hyperplastic breast tissue when cultured in the 3D BME system.

### Int-*β*3 expression in MCF-7 tumors promotes their differentiation in a hESC-based teratoma model

We tested whether expression of Int-*β*3 in MCF-7 cells will promote their differentiation *in vivo* within the microenvironment of normal differentiated human cells, given that tumor microenvironment has been shown to greatly influence tumorigenicity properties. For this purpose, we generated human teratomas derived from hESC in SCID/beige mice, which comprised a wide variety of non-transformed differentiated tissues of human origin.^13, 14^ After 6 weeks either MCF-7-Int*β*3-GFP cells or MCF-7-vec-GFP cells were injected into mature teratomas. Teratomas bearing tumors were collected after 3–4 weeks and paraffin sections were prepared and stained with H&E or subjected to immunohistochemistry using anti-Int*β*3 or anti-GFP antibodies. Immunohistological analysis revealed that MCF-7-vec-GFP cells, initially originating from pleural effusion of human breast adenocarcinoma, similarly generated invasive tumors when inculcated into the teratomas. Whereas, inoculated MCF-7-Int*β*3-GFP cells generated more differentiated tumors depicted by formation of tubular structures with lumens, significant reduction in nuclear pleomorphism, less hyper chromatic nuclei and lower mitotic figures (Figure 6e and Supplementary Figure S7C-D). Taken together, these results suggest that MCF-7-Int*β*3 tumors grown in the human teratoma microenvironment were more differentiated compared with MCF-7 tumors.

### Int-*α*V*β*3 promotes downregulation of Notch4 signaling

**Figure 7:**
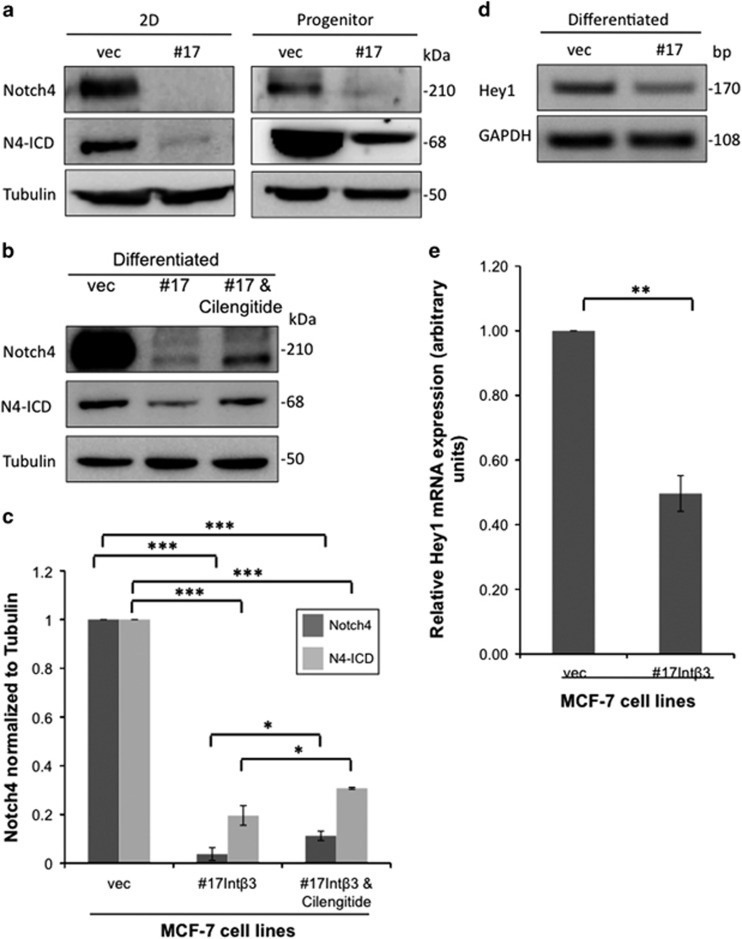
**I**nhibition of Notch4 signaling in differentiating MCF-7-Int*β*3 cells. (**a**–**b**) W.B. analysis and (**c**) quantification of Notch4 and N4-ICD expression in **a**. Lysates collected either directly from 2D culture (2D) or from CLPs derived from 2D culture of MCF-7-vec and MCF-7-Int*β*3 (progenitor) and after their differentiation in the 3D BME system (**b**). (**c**) Quantification of Notch4 (*n*=3) and N4-ICD expression (*n*=2) normalized to tubulin. (**d**) Semi-qPCR analysis of mRNA transcript levels of Hey1 in cells cultured in the 3D BME system. (**e**) qPCR quantification of Hey1 mRNA levels; values were normalized to GAPDH (*n*=2) (*P⩽0.05; **P⩽0.05 and ***P⩽0.001)

**Figure 8:**
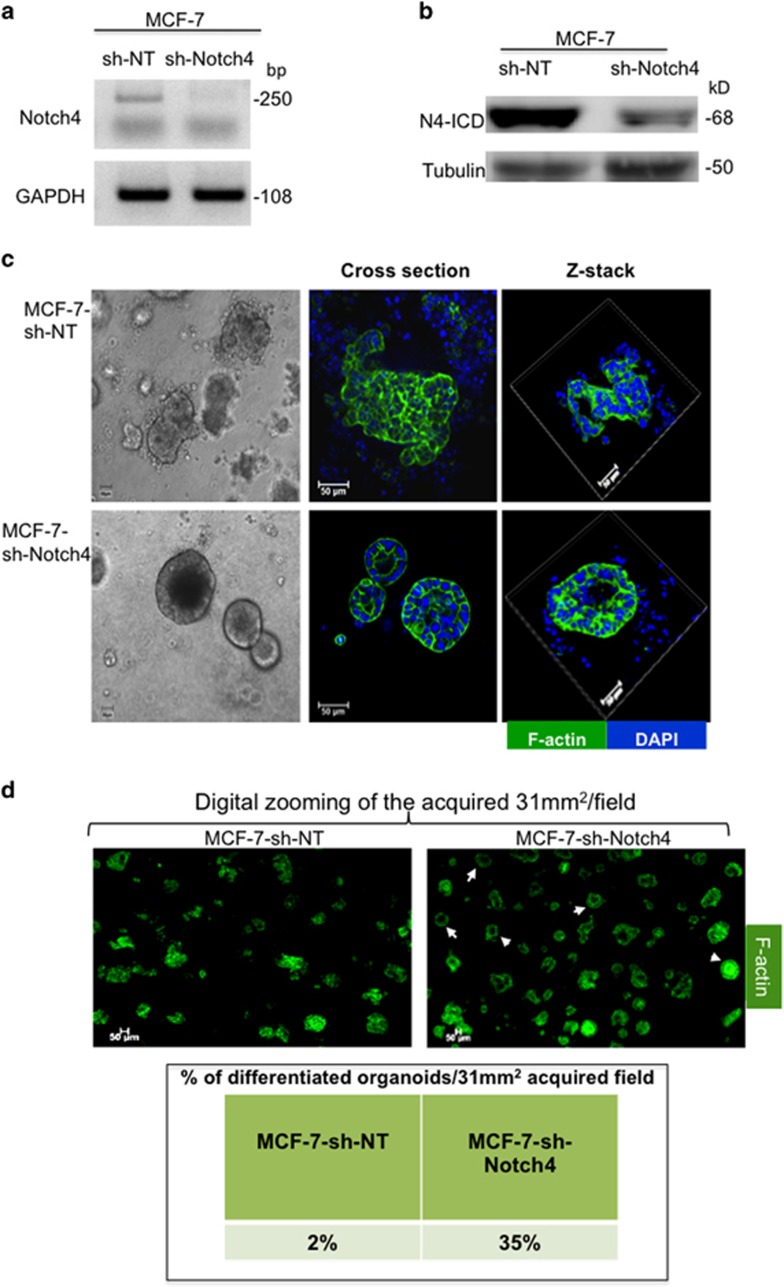
Knockdown of Notch4 expression in MCF-7 cells promotes their differentiation into acinar-like organoids in the 3D BME system. Cells derived from 2D culture of MCF-7-sh-non-target (sh-NT) and MCF-7-sh-Notch4 cell lines. (**a**) Semi-qPCR analysis for the knockdown of Notch4 expression. (**b**) W.B. analysis for the knockdown of N4-ICD expression. (**c**–**d**) MCF-7-sh-NT and MCF-7-sh-Notch4 cells cultured in the 3D BME system for 32 days. (**c**) Left panel: representative light microscopy images (magnification × 20). Middle panel: representative confocal images of the cross section through the middle of the organoids. Right panel: representative confocal images of z-stacks of organoids stained with Dapi for nuclei (blue), F-actin (green). Magnification × 40. Bars=50 *μ*m. (**d**) Top panel: representative confocal images of the cross section through the middle of a magnified portion of an acquired image at 31 mm^2^/field stained with F-actin (green). White arrowheads indicate differentiated organoids. Magnification × 10. Bars=50 *μ*m. Bottom panel: table representing percentage of differentiated organoids/31 mm^2^ acquired field

Previous studies implicated the role of Notch4 signaling in cell fate, such as differentiation of progenitor cells and tumorgenesis, and that activation of *Notch4* gene inhibited mammary epithelial cell differentiation and promoted tumor formation.^29, 30, 31^ This has prompted us to test whether differentiating CLP-Int-αv*β*3^pos^ cells will inhibit Notch4 expression and its downstream signaling in the 3D BME system. Importantly, Notch4 is activated upon its interaction with its ligands leading to Notch4 cleavage. This results in release of the active intracellular form of Notch4 (N4-ICD), which translocates to the nucleus regulating transcription of targeted genes such as *Hey1*.^32^ Therefore, we flow sorted either the CLP-Int-αv*β*3^neg^ or CLP-Int-αv*β*3^pos^ (as described in Figure 4a). Protein lysets were extracted from CLPs before culturing them in the 3D BME system (designated progenitors, Figure 7a) and after their differentiation in the 3D BME system (designated differentiated, Figure 7b) once acini were apparent (at day 30–40). In addition, protein was extracted from the total subpopulations of cells derived from MCF-7-vec and MCF-7-Int*β*3 #17 cells grown in 2D culture (designated 2D, Figure 7a). Our results demonstrate that both the Notch4 and N4-ICD were highly expressed in MCF-7-vec cells in 2D culture and in flow sorted CLP-Int-*α*v*β*3^neg^, before and after their culture in the 3D BME system. Whereas, Int-αv*β*3 expression in both MCF-7 cells grown in 2D culture and in CLPs inhibited the expression of both Notch4 and N4-ICD (Figures 7a and b). This significant inhibition was maintained after their differentiation in the 3D BME system (Figures 7b and c) and was partially reversed by inhibiting Int-αv*β*3 activity with cilengitide (Figures 7b and c). To further evaluate the downstream signaling of Notch4, we looked for the transcription regulation of Hey1. Our results demonstrates a 50% significant reduction in Hey1 mRNA levels in differentiating acinar-like organoids compared with that in non-differentiating organoids in the 3D BME system (Figures 7d and e). Overall, these results suggest that differentiation of CLP-Int−αv*β*3^pos^ to normal acinar-like organoids in the 3D BME system is mediated by downregulation of Notch4 signaling. Indeed, stable knockdown of Notch4 and its activated form (N4-ICD) in MCF-7 cells by shRNA targeting of Notch4 expression (Figures 8a and b) was sufficient to promote their differentiation in the 3D BME system (Figures 8c and d).

## Discussion

Our results demonstrate that expression of Int-αv*β*3, previously shown to play a role in tumor progression (reviewed in ref. 33), can surprisingly promote differentiation of CLPs^low^ in conjunction with their microenvironment into (i) a precancerous stage resembling human UDH when placed in the 3D BME system (ii) and to a more differentiated phenotype when introduced *in vivo* into a supportive microenvironment of human origin. Furthermore, our results suggest that Int-*α*v*β*3 may mediate this reversion by downregulating Notch4 signaling.

Ectopic expression and activation of Int-*α*v*β*3 in luminal A breast cancer cell lines preferentially expanded the prominent CLPs^low^ population when grown only as non-adherent mammospheres. Similarly, the presence of EpCAM^pos^CD49f^pos^ (CLPs) subsets of cells in luminal A and B breast cancer cell lines and tissues were reported previously.^15^ Interestingly, Int-*α*v*β*3 re-expression in luminal A breast cancer cell lines did not expand the CSCs population as was reported for other types of cancers,^34^ thus suggesting that Int-*α*v*β*3 diverse effect depends on cancer type and subtype.

Here we demonstrate that re-expression of Int-*α*v*β*3 will promote luminal A breast cancer cells to differentiate into polarized acinar-like organoids containing a hollow lumen when cultured in the 3D BME system. This differentiation was not dependent on the expansion of CLPs^low^ by Int-*α*v*β*3 expression, but rather was dependent on their commitment to differentiate via Int-*α*v*β*3 expression. Moreover, Int-*α*v*β*3 inhibition by cilengitide significantly reduced the number of differentiated acini. Importantly, the formation of the acini was mediated by cellular death due partly to apoptosis, as reported during the normal morphogenesis of mammary acini.^25, 26, 27^ In addition, our results demonstrate that these differentiated acini retain their luminal phenotype (expressing ER, GATA3 and MUC-1) and surprisingly displayed an alveolar phenotype with milk protein *β*-casein expression confined to the outer polarized layer of cells surrounding the hollow lumen with no lactogenic stimulus. Interestingly, a recent report demonstrated that Int-*β*3 is required for mouse mammary development during pregnancy.^35^ However, in that model system Int-*β*3 expression was required for the expansion of the pregnancy-associated mammary stem cells.

Evidence supports the idea that disruption of cell-polarity mechanisms promotes tumor initiation, thus suggesting a role of cell and tissue polarity mechanisms as potential non-canonical tumor suppressors.^36, 37^ Several studies demonstrated how interfering with adhesion molecules that are aberrantly expressed in the cancer cells and the microenvironmental context can redirect breast cancer cells to a normal-like phenotype.^12, 21, 38, 39, 40, 41^ Importantly, this reversion was associated with growth arrest, similar to normal mammary tissue.^26, 38^ Concordantly, our results demonstrate that the reversion of Int-*α*v*β*3 expressing cells to normal-like acini culminated in their growth arrest.

Similarly, a previous *in vivo* study demonstrated that MCF-7-Int*β*3 cells developed very small indolent tumors compared with MCF-7 cells.^42^ To further characterize the behavior of MCF-7-Int*β*3 cells in the context of their microenvironment, we utilized a tumor microenvironment model on the basis of the potential of hESC to generate teratomas in immunodeficient mice. This model provides an *in vivo* humanized tumor microenvironment.^13^ Intriguingly, we demonstrated that Int-*β*3 expression in MCF-7 cells in conjunction with the teratoma microenvironment developed more differentiated tumors compared with MCF-7-vec cells, however, both cell lines retained their invasive properties as was evident by histology and presence of dormant disseminated tumor cells in the lungs (data not shown). Taken together, our results demonstrate that the composition of the tumor microenvironment engaging with MCF-7-Int*β*3 cells will dictate the extent of their differentiation. Furthermore, our findings suggest that complete halt of tumor progression may require targeting the signaling pathways that mediate invasion concomitantly with those promoting differentiation; such as those emanating from Int-*α*v*β*3.

Examination of the mechanisms responsible for this differentiation revealed that Int-*α*v*β*3 expression inhibited Notch4 and N4-ICD expression and downstream signaling in differentiating CLPs^low^ derived from MCF-7 cells. Conversely, inhibiting the function of Int-*α*v*β*3 with cilengitide partially restored Notch4 expression and activation leading to significant reduction in the number of differentiated organoids. Hence, these results suggest that inhibiting Notch4 in CLPs^low^ will promote their differentiation into a normal acinar-like organoids in the 3D BME system. Indeed, we demonstrate that knockdown of Notch4 expression in MCF-7 cells was sufficient to promote their differentiation in the 3D BME system. These findings are in concordance with previous reports demonstrating that overexpression of the activated Notch4 oncoprotein in normal breast epithelial cells abrogated their normal morphogenesis in 3D culture.^43^ Furthermore, knockdown of Notch4 in MCF-7 cells completely abolished their ability to form tumors in the mouse mammary fat pad.^44^ Overall, the results described above suggest that Int-*α*v*β*3 expression in CLPs^low^ will promote their differentiation into acinar-like organoids via downregulation of Notch4 expression and downstream signaling, thus inhibiting tumor growth.

The reversion of the malignant phenotype to a more differentiated or benign phenotype is the goal of differentiation therapy and if successful, may change the prognosis of most patients with recurrent cancer by decades. We demonstrated that the acini arising from CLP-Int-*α*v*β*3^pos^ in the 3D BME system resemble UDH – a benign stage of breast tissue. Intriguingly, Int-*α*v*β*3 is shown to be selectively expressed in the epithelium of UDH of breast tissues, and is lost during early stages of luminal A breast cancer progression. Whereas, in the late stage Int-*α*v*β*3 expression was mostly confined to the stroma, the latter previously shown to play a role in cancer progression.^45^ These surprising results differ from previous reports suggesting that tumoral expression of Int-*α*v*β*3 plays a role in tumor progression (reviewed in ref. 33). Importantly, we have also conducted a meta-analysis of 3992 breast cancer patient samples previously developed (Pubmed ID PMID: 23964924) and demonstrated that Int-*β*3 expression levels cannot predict overall survival and disease-free survival of luminal A breast cancer patients (Supplementary Figure S8). These results may well reflect our observation of the spatial expression (epithelium *versus* stromal cells) and temporal expression of Int-*β*3 in different stages of luminal A breast cancer progression and suggest that our observations are specific to luminal A breast cancer subtypes.

Taken together, our study demonstrates that CLPs of luminal A breast cancer cells can be induced to differentiate to a more benign phenotype in conjunction with their microenvironment by promoting downstream signaling that emanates from Int-*α*v*β*3, such as inhibition of Notch4 signaling. Promoting differentiation of luminal A breast cancer cells by targeting Notch4 signaling may be an attractive target given Notch4-restricted expression in normal tissue.^46^ Development of selective monoclonal antibodies and/or potential use of luteolin, recently shown to inhibit Notch4 signaling^47^ should be considered for future treatment of recurring and/or endocrine-resistant breast disease. Such an approach may provide the means to maintain a chronic and dormant-like state of the recurring breast disease and will hopefully extend patients survival with minimal side effects.

## Materials and Methods

### Cell lines culture and reagents

MCF-10A cell were obtained from Dr. Israel Vlodavsky (Technion Ins, Haifa, Israel) and were maintained as described previously.^48^ MCF-7 and T47D cells were obtained from American Type Culture Collection (ATCC) and were stably transected with pCDNA3-Int*β*3 (a gift from Dr. Newman PJ; The Blood Research Center of Southeastern Wisconsin Inc.). Clones of MCF-7-Int*β*3 and pool of T47D-Int*β*3 were selected by G418 (Gold Biotechnology, St. Louis, MO, USA) and were maintained in DMEM or RPMI supplemented with 10% fetal bovine serum and antibiotics (Life Technologies, Hertzliya Pituach, Israel), respectively. 3D cultures were carried out in growth factor-reduced Cultrex Basement Membrane Extract (Trevigen, Inc., Gaithersburg, MD, USA) ^49^ as previously described.^48, 50^ Cilengitide, was a kind gift from Dr. Ronit Satchi Fainaro (University of Tel Aviv). Immunofluorescent images were captured by either Zeiss LSM 700 or by Nikon A1R confocal microscope.

### Mammosphere assay

The different MCF-7 and T47D cell lines were dissociated into single cells and plated on ultralow attachment 24-well plates (Corning) (2000 cells/ml) as described previously.^21^ Quantification of the number of mammospheres was done by light microscopy at magnification × 10 for counting all mammospheres/field. Experiments were repeated three times with three replicates each.

### Flow cytometric analysis (FACS)

Single cells were dissociated and were characterized for Int-*α*v*β*3^−/+^ expression (using anti-CD51/CD61-PE) and the gated populations were further characterized for the expression of EpCAM (using anti-EpCAM-APC), CD49f (using anti-CD49f-FITC) and CD24 (anti-CD24-PerCP) using FACSCanto II (BD). For CSC characterization: CD44^high^ (using anti-CD44-Brilliant Violet 421^TM^) and CD24^low^ (using anti-CD24-PerCP) expression was determined. All antibodies were obtained from Biolegend (San Diego, CA, USA).

### Quantitative RT-PCR

RNA was reversed-transcribed using the High Capacity RNA-to-cDNA Kit (Applied Biosystems, Carlsbad, CA, USA). The cDNA was used as a template for semi-quantitative and for quantitative PCR using the PCR Dream Taq Mix (Thermo-scientific, Waltham, MA, USA) or the Fast SYBR Green Master Mix kit, respectively. Analysis of gene expression was performed with the StepOneTM and StepOnePlusTM Real-Time PCR detection system (Applied Biosystems) using the relative standard curve method. The following PCR primers (forward and reverse, respectively) for human GAPDH, GATA3 and N*o*tch4 were designed using the Integrated DNA Technologies Inc software. Human GAPDH: 5′-ATGGGGAAGGTGAAGGTCG-3′ and 5′-GGGGTCATTGATGGCAACAATA-3′ Human GATA3: 5′-GCCCCTCATTAAGCCCAAG-3′ and 5′-TTGTGGTGGTCTGACAGTTCG-3′ Human ER: 5′-AAGAGCTGCCAGGCCTGCC-3′ and 5′-TTGGCAGCTCTCATGTCTCC-3′^51^ Human Hey1: 5′-TGAGCTGAGAAGGCTGGTAC-3′ and 5′-ACCCCAAACTCCGATAGTCC-3′^52^ Human *β*-casein: 5′-CCCTCAAATCCCAAAACTCA-3′ and 5′-GAGCAGAAGGGCTTGAACAG-3′^53^ Human Notch4: 5′-GATGGGCTGGACACCTACAC-3′ and 5′-CACACGCAGTGAAAGCTACCA-3′.

### Short hairpin RNA silencing experiments

MCF-7 cells were infected with Mission shRNA lentiviral particles targeting either human Notch4 (sh-Notch4) (Clone ID#TRCN0000426949) or with non-target shRNA (sh-NT) (Sigma Aldrich Israel, Rehovot, Israel), selected with puromycin (2 *μ*g/ml; Sigma) and maintained in DMEM supplemented with 10% fetal bovine serum and antibiotics (Life Technologies).

### Immunoblot

Immunoblots were conducted as previously described.^54^ Specifically, cells grown in 3D BME were extracted with ice cold PBS supplemented with 5 mM EDTA (1.5 h at 4°C on a shaker). Pellets were lysed in WCE (whole-cell extract) buffer (25 mM Hepes, pH 7.7, 0.3M NaCl, 1.5 mM MgCl_2_, 0.2 mM EDTA, 0.1% Triton X-100, 100 *μ*g/ml PMSF and protease inhibitor cocktail (Roche, 1 : 100 dilution)). The proteins were separated by SDS-PAGE (8/10/12%) followed by transfer on to a nitrocellulose membrane. The membranes were blocked with either 5% (w/v) non-fat dried skimmed milk powder in PBS supplemented with 0.05% Tween20 (PBS-T) or with 5% BSA in TBST for 1 h at room temperature. Membrane was then probed either with mouse anti-human GATA3 (1 : 500; Biolegend), mouse anti-human ER (1 : 200), rabbit anti-human P21 (1 : 1000), rabbit anti-*β*-casein (1 : 200), rabbit anti-tubulin (1 : 1000; Santa Cruz, Dallas, TX, USA), rabbit anti-human Notch4 (1 : 400; Abcam, Cambridge, MA, USA), at 4°C overnight. Next, Horseredish peroxidase-conjugated secondary antibodies to rabbit or mouse immunoglobulin G (IgG) were used (1 : 10 000; Jackson ImmunoResearch Laboratories, West Grove, PA, USA) for 1 h at room temperature and washed 15 min × 3 with PBS-T. Western Bright ECL (Advansta, Menlo Park, CA, USA) was added to the membrane for 30 s and analyzed using ImageQuant LAS-4000 analyzer (GE Healthcare Life Sciences, Pittsburgh, PA, USA) and ‘ImageQuant LAS-4000' software (GE Healthcare Life Sciences). Notably, Notch4 and N4-ICD detection was obtained by 15 and 4–5 min exposure, respectively. Densitometry analysis was performed using ImageQuant total lab-7 (GE Healthcare Life Sciences) image analysis software.

### Immunofluorescence staining in 3D culture

Immunofluorescence staining for MUC-1, Laminin-5, *β* casein and Ki67 was carried out as described previously^54^ with some modifications. The different cell lines, 7 × 10^3^ cells/well on the eight-chamber glass slide system were cultured in Cultrex growth factor-reduced Basement Membrane Extract (BME: Trevigen, Inc) as described previously.^48, 50^ Fixed cells were blocked with either 3% BSA or IF buffer (130 mM NaCl, 7 mM Na_2_HPO_4_, 3.5 mM NaH_2_PO_4_, 7.7 mM NaN_3_, 0.1% BSA, 0.2% Triton X-100, 0.05% Tween20) supplemented with 10% donkey serum for 1 h and incubated overnight at 4 °C with either antibody. The primary antibodies used were as follows: rabbit monoclonal antibody to Ki67 (conjugated with FITC; 1:100), rabbit polyclonal antibody to laminin 5 (1 : 500), rabbit antibody to MUC-1 (1 *μ*g/ml) and rabbit antibody to *β*-casein (5 *μ*g/ml) from Abcam. The cells were washed three times with PBS for 15 min each, and incubated for 60 min with donkey anti-rabbit conjugated to Alexa Fluor 647 (Invitrogen, Carlsbad, CA, USA), washed as above, and mounted with VECTASHIELD mounting medium with 4′, 6-diamidino-2-phenylindole (DAPI). For F-actin staining, cells were incubated overnight with Alexa-Fluor 488 Phalloidin (1 : 40) (Molecular Probes, Eugene, OR, USA), washed three times with PBS for 15 min each and mounted with VECTASHIELD mounting medium with DAPI. Immunofluorescent images were captured by either Zeiss LSM 700 confocal laser scanning microscope or Nikon A1R confocal microscope.

### Paraffin biopsies

Paraffin biopsies from normal patients and from patients with different stages of luminal A breast cancer were obtained from the Institute of Pathology, Johannes Gutenberg University, Mainz. The local ethical review board approved use of the tissue samples. Immunohistochemical staining for Int-*β*3 expression was carried out on the paraffin sections as described below.

### Teratoma and tumor formation

SCID/beige mice were purchased from Harlan Laboratories Ltd., Israel. The mice were housed under specific pathogen-free conditions. The experimental protocols were approved by the committee for cversight of animal experimentation at the Technion – Israel Institute of Technology, Haifa, Israel.

Teratoma formation was carried out as previously described.^13^ Briefly undifferentiated hESC clone H9.1 (46XX) were injected into the hindlimb musculature of SCID/beige mice (~5 × 10^6^ cells per injection). The formed teratoma was composed of a wide variety of disorganized but normal differentiated human tissue and structures, comprising differentiated cell types representing derivatives of all three major embryonic lineages.^55^ At 6–7 weeks following initial injection of hESC, 4 × 10^6^ of either MCF-7-vec-GFP or MCF-7-Intβ3-GFP cells were injected into the teratoma and were allowed to grow for an additional 3–4 weeks. Teratomas were collected and prepared for paraffin sections and immunohistochemistry staining for Int-*β*3 and GFP expression was carried out as described below.

### Immunohistochemical staining

Paraffin blocks were sectioned at 4 mm thickness and were microwave pretreated in citrate buffer (pH 6.0) for antigen retrieval. Endogenous peroxidase activity was blocked using 3% H_2_O_2_ in methanol. Sections were washed and blocked with goat serum for 1 h and incubated with either the primary antibody for Int-*β*3 (1 : 250 for biopsies sections and 1 : 50 for tetatoma sections; Abcam) or with primary antibody for GFP (1 : 500; Abcam) overnight at 4 °C. Immunohistochemical detection was performed using the labeled streptavidin biotin complex method (Histostain Plus Bulk Kit; Zymed Laboratories, Inc., San Francisco, CA). AEC (3-amino-9-ethylcarbazole) was used as a chromogen, and slides were counterstained with hematoxylin. The biopsies sections were scored for the frequency and the intensity of Int-*β*3 staining by an expert pathologist at Ramba Medical Center. The frequency of staining was determined by counting the percentage of positive staining of epithelial cells for Int-*β*3 expression per cluster and the intensity of staining was evaluated ranging from 0–3.

### Histomorphometry

Fluorescent and bright field images of the breast organoids were analyzed with the Image Pro Plus 7 (Media Cybernetics, MA, USA). The bright field images were measured for the total area of the breast tissue organoids in Microns Square and their contour irregularity (colony roundness=colony perimeter^2^/(4 x pi x colony area)). The area and the roundness are built in parameters that were provided by the commercial program (Image Pro Plus). The Image Pro Plus software automatically counted the DAPI images after finding the appropriate threshold of the colored pixels. Only single intact nuclei were scored (the range of nuclei size that was scored was ≥7⩽160). An average number of 255–324 nuclei were analyzed per cluster. The cells counts were expressed as number of epithelial cells per acinus.

### Statistical analysis

Student's unpaired *t-*test was used for data analysis. Comparison of the histomorphometric parametric variables between two groups was done using unpaired student *t*-test after evaluating the variances using the Leven's test. Two tailed *P-*values of 0.05 or less were considered to be statistically significant.
