# Supplementary material for: ‘Normalizing' the malignant phenotype of luminal breast cancer cells via alpha(v)beta(3)-integrin
Source: Cell Death Dis. 2016 Dec 1;7(12):e2491–. doi: 10.1038/cddis.2016.387 (PMC5260995; doi:10.1038/cddis.2016.387)
Supplement: Supplementary Figures [file cddis2016387x1.pdf]

A

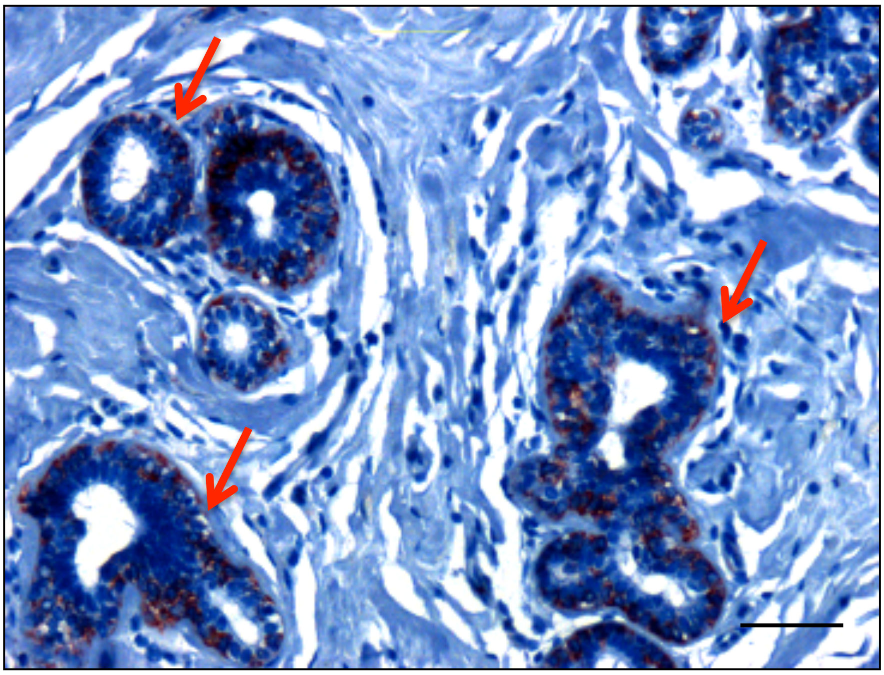

B

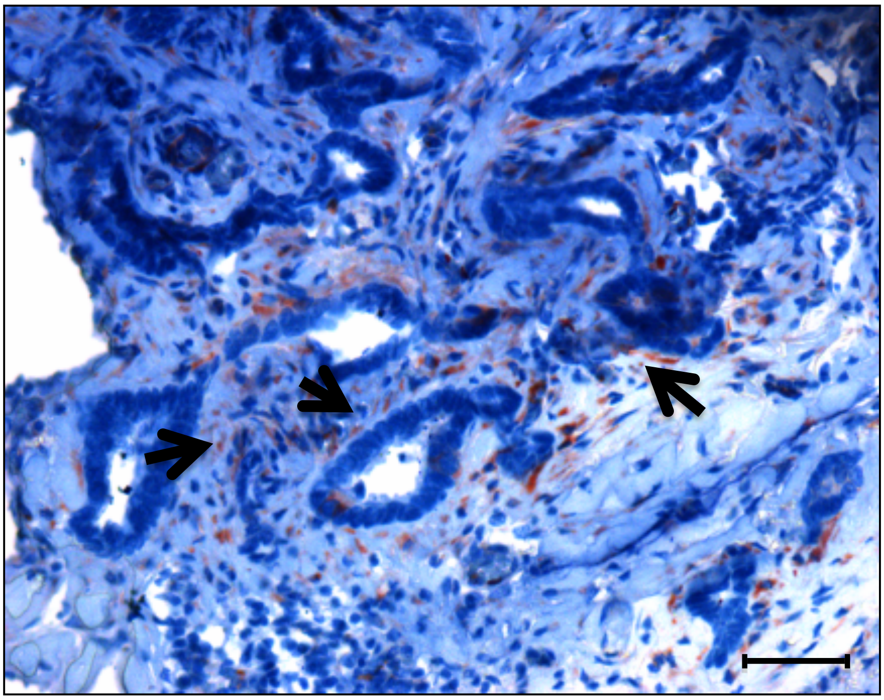

Fig. S1

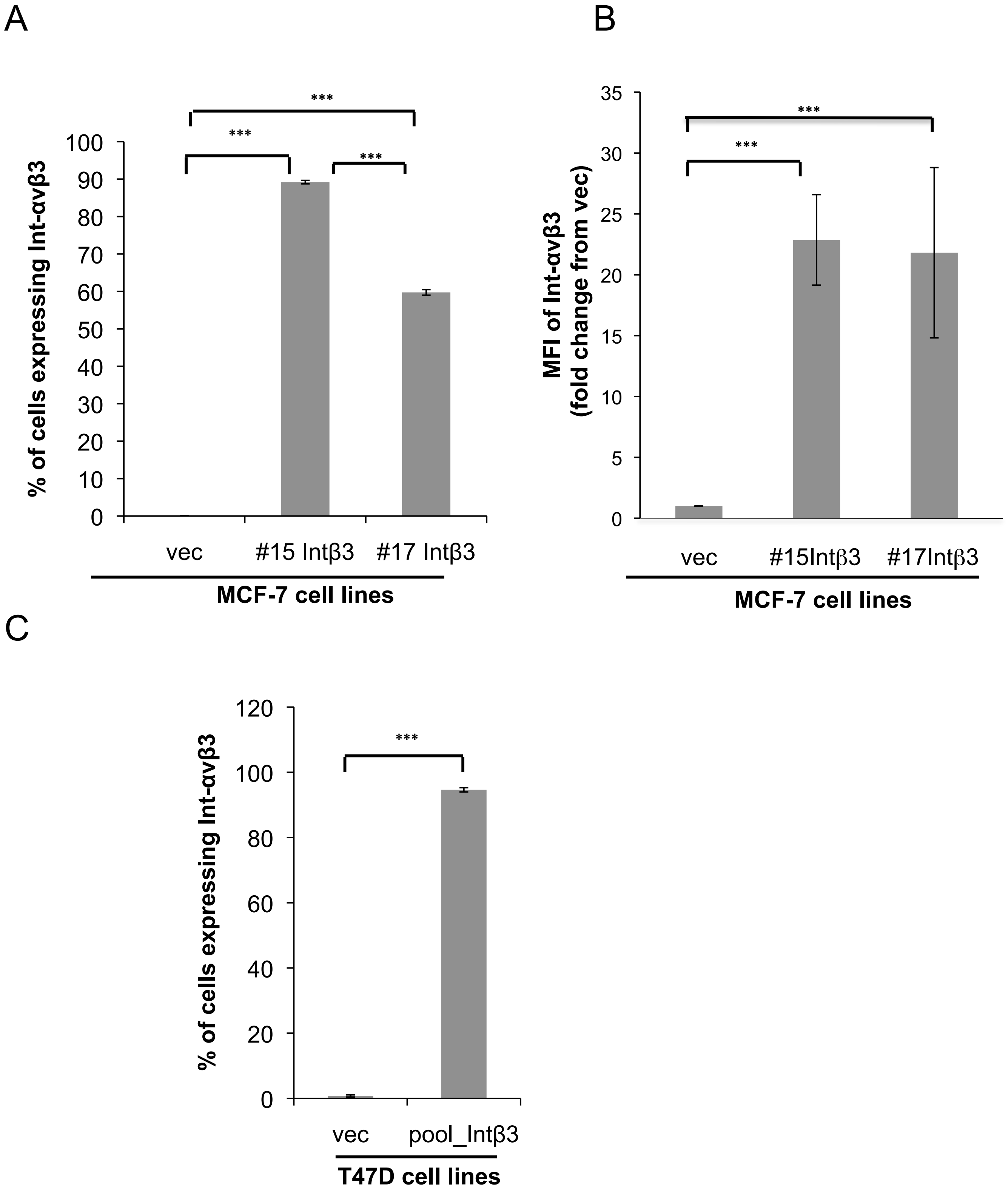
A
B
C
% of cells expressing Int-αvβ3
100
90
80
70
60
50
40
30
20
10
0
***
***
***
vec
#15 Intβ3
#17 Intβ3
MCF-7 cell lines
MFI of Int-αvβ3
(fold change from vec)
35
30
25
20
15
10
5
0
***
***
vec
#15Intβ3
#17Intβ3
MCF-7 cell lines
% of cells expressing Int-αvβ3
120
100
80
60
40
20
0
***
vec
pool_Intβ3
T47D cell lines

Fig. S2

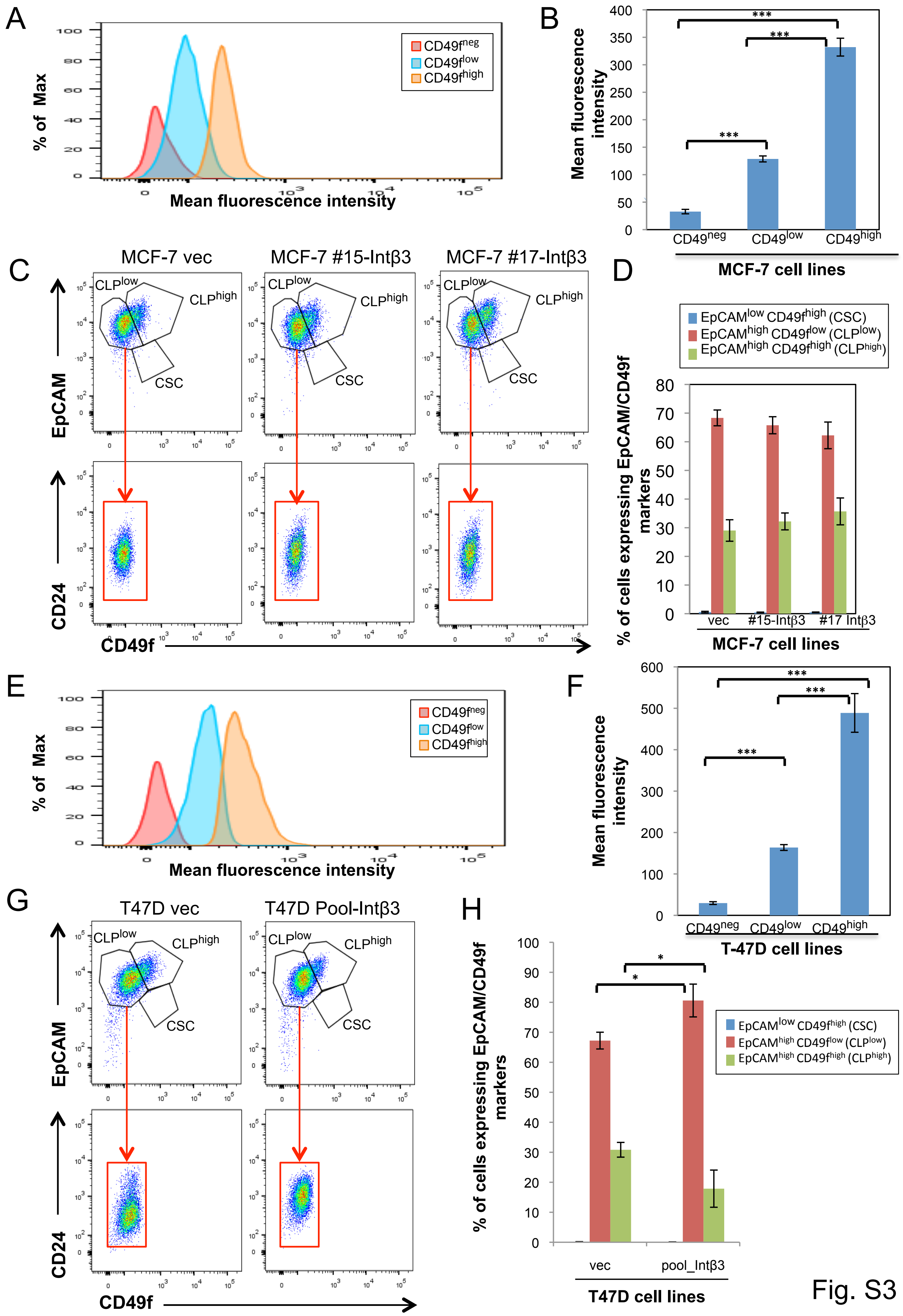
A
% of Max
Mean fluorescence intensity
CD49f neg
CD49f low
CD49f high
B
Mean fluorescence intensity
CD49neg
CD49low
CD49high
MCF-7 cell lines
C
MCF-7 vec
MCF-7 #15-Intβ3
MCF-7 #17-Intβ3
CLPlow
CLPhigh
CSC
EpCAM
CD24
CD49f
D
EpCAMlow CD49fhigh (CSC)
EpCAMhigh CD49flow (CLPlow)
EpCAMhigh CD49fhigh (CLPhigh)
% of cells expressing EpCAM/CD49f markers
vec
#15-Intβ3
#17 Intβ3
MCF-7 cell lines
E
% of Max
Mean fluorescence intensity
F
Mean fluorescence intensity
T-47D cell lines
G
T47D vec
T47D Pool-Intβ3
H
% of cells expressing EpCAM/CD49f markers
vec
pool_Intβ3
T47D cell lines

Fig. S3

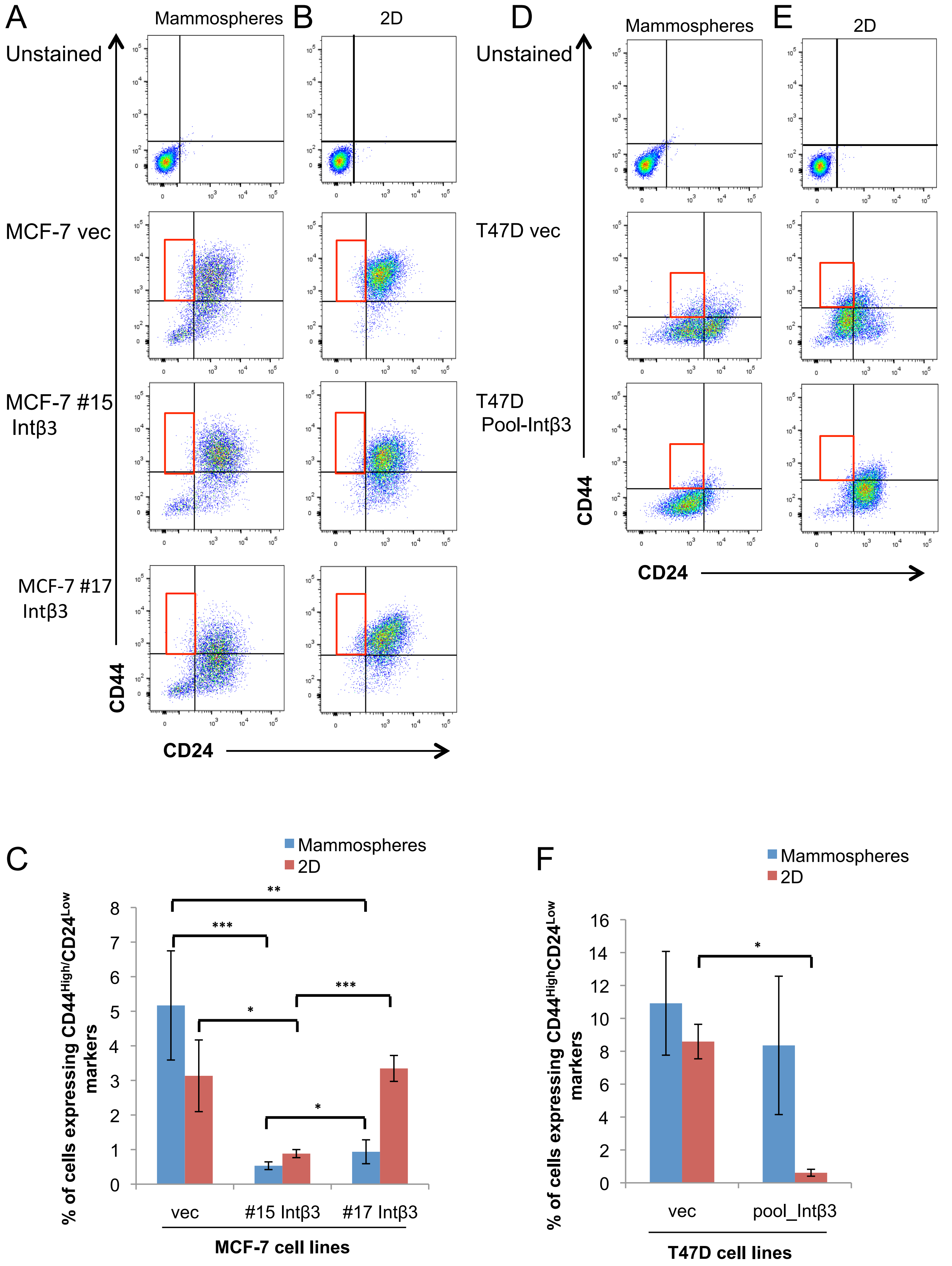
A
Mammospheres
B
2D
Unstained
MCF-7 vec
MCF-7 #15
Intβ3
MCF-7 #17
Intβ3
CD44
CD24
D
Mammospheres
E
2D
Unstained
T47D vec
T47D
Pool-Intβ3
CD44
CD24
C
Mammospheres
2D
% of cells expressing CD44High/CD24Low markers
vec
#15 Intβ3
#17 Intβ3
MCF-7 cell lines
F
Mammospheres
2D
% of cells expressing CD44HighCD24Low markers
vec
pool_Intβ3
T47D cell lines

Fig. S4

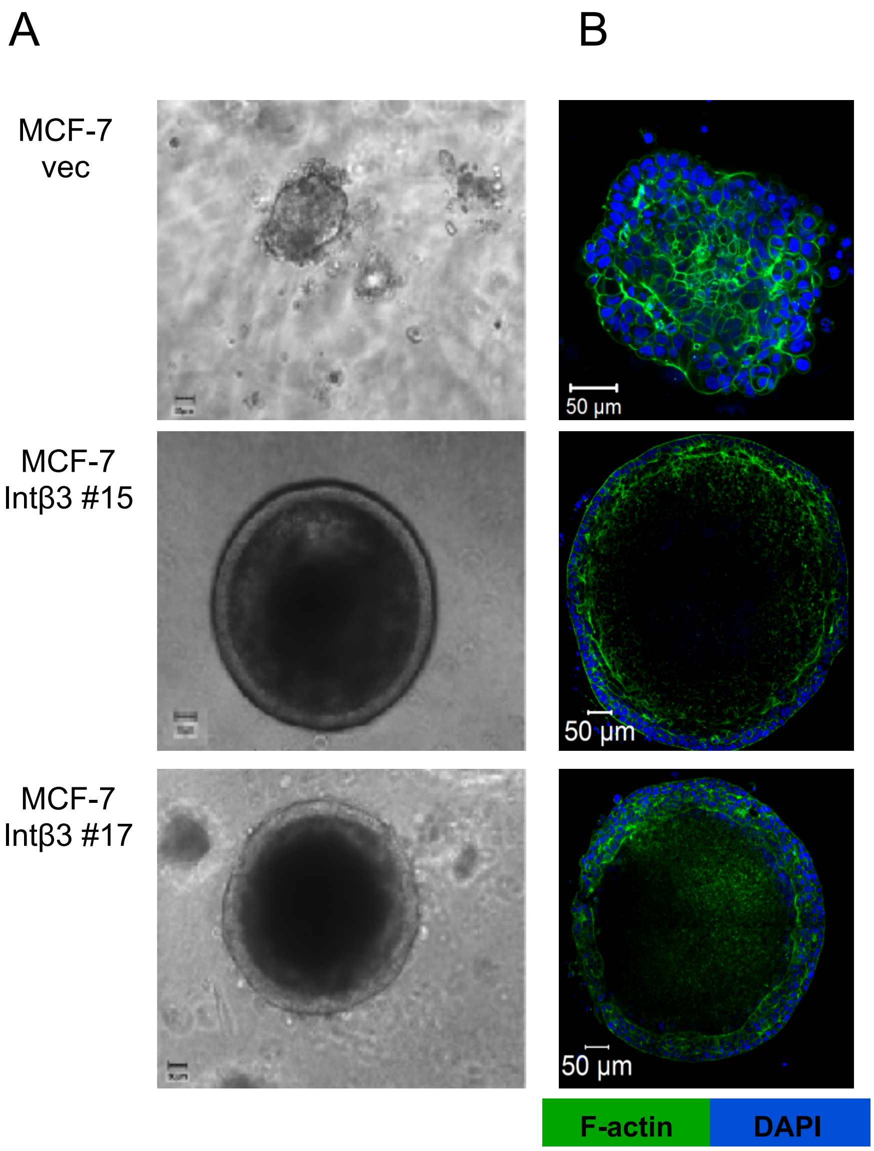
A
B
MCF-7
vec
MCF-7
Intβ3 #15
MCF-7
Intβ3 #17
50 µm
50 µm
50 µm
F-actin
DAPI

Fig. S5

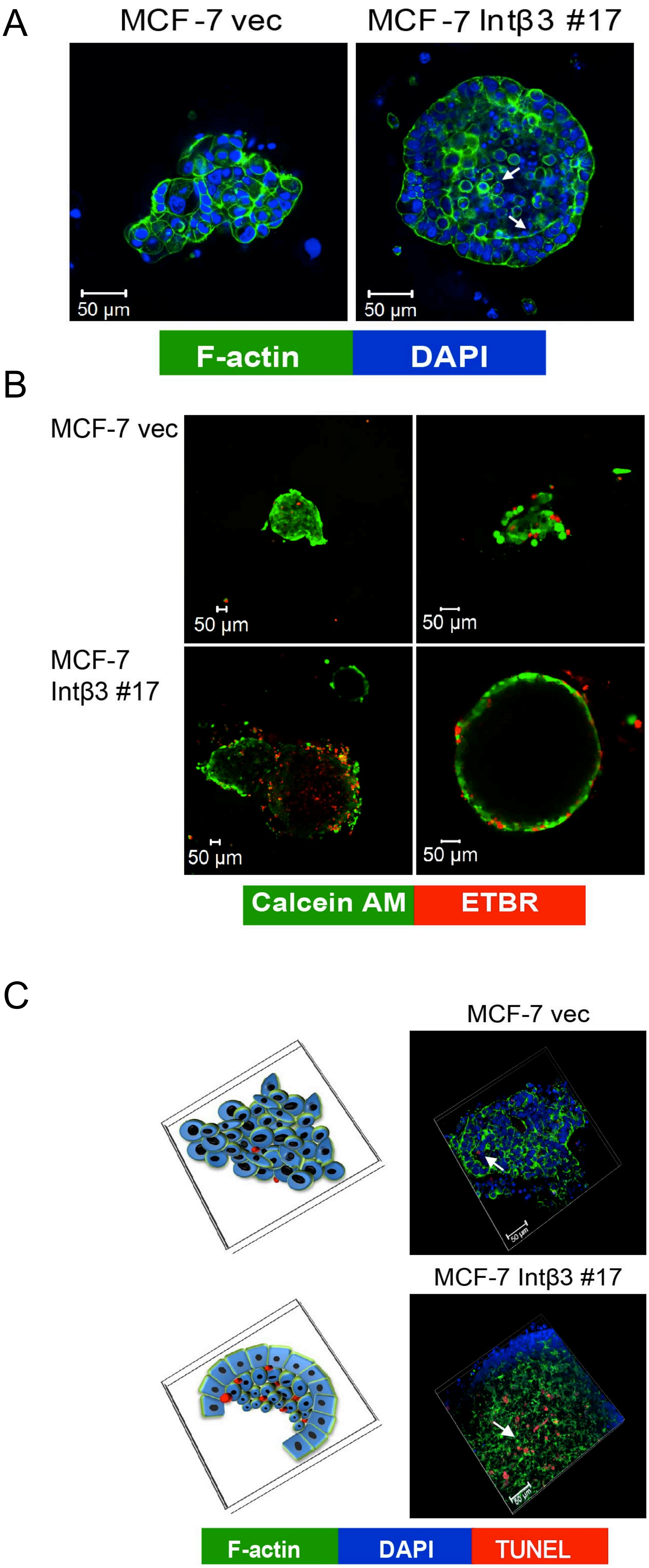
A
MCF-7 vec
MCF-7 Intβ3 #17
50 μm
50 μm
F-actin
DAPI
B
MCF-7 vec
MCF-7 Intβ3 #17
50 μm
50 μm
50 μm
50 μm
Calcein AM
ETBR
C
MCF-7 vec
MCF-7 Intβ3 #17
F-actin
DAPI
TUNEL

Fig. S6

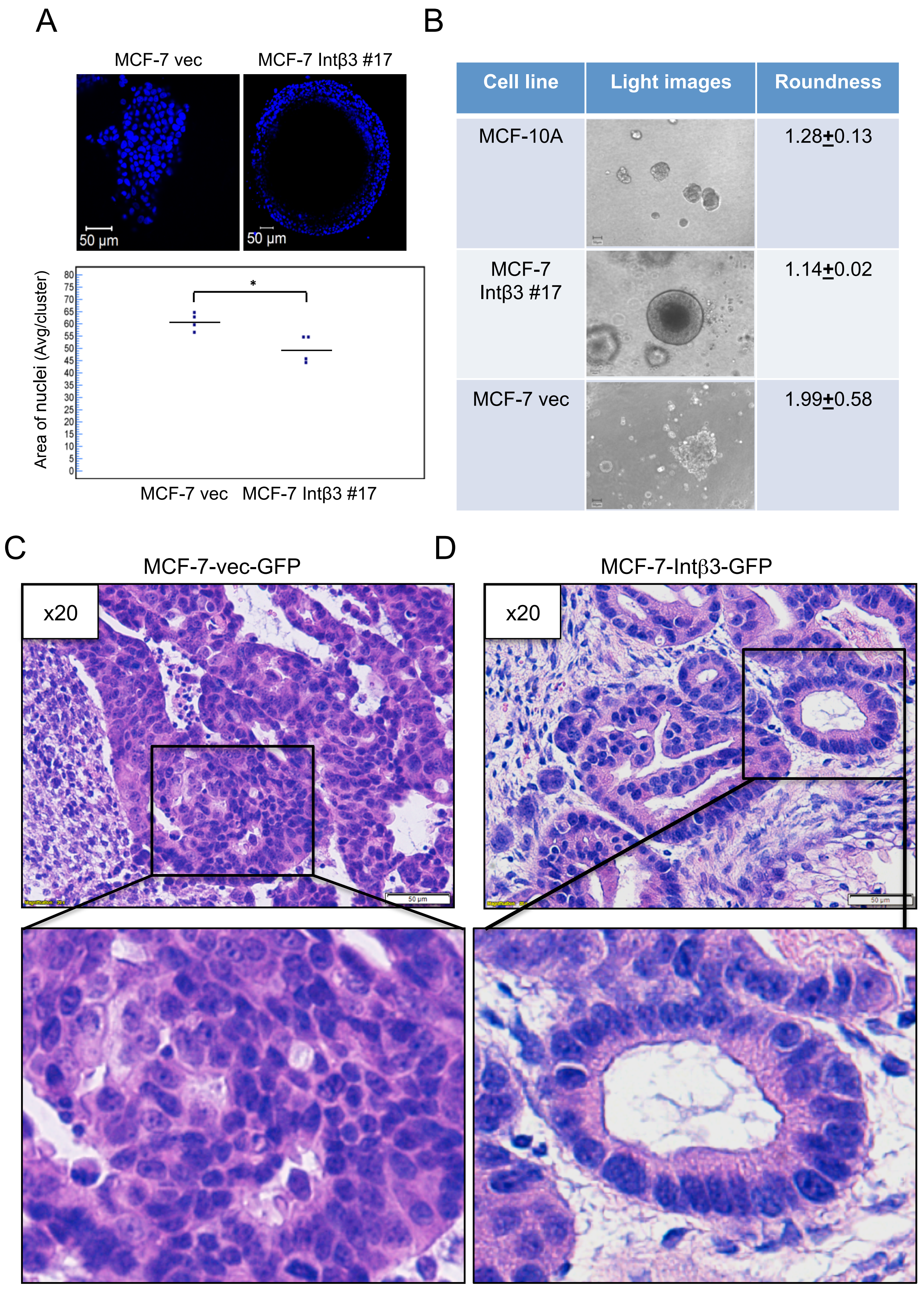
A
MCF-7 vec
MCF-7 Intβ3 #17
50 μm
50 μm
*
Area of nuclei (Avg/cluster)
0 5 10 15 20 25 30 35 40 45 50 55 60 65 70 75 80
MCF-7 vec
MCF-7 Intβ3 #17
B
Cell line
Light images
Roundness
MCF-10A
1.28±0.13
MCF-7 Intβ3 #17
1.14±0.02
MCF-7 vec
1.99±0.58
C
MCF-7-vec-GFP
x20
D
MCF-7-Intβ3-GFP
x20

Fig. S7

Intβ3– Luminal A

Overall Survival (n = 357)
Median

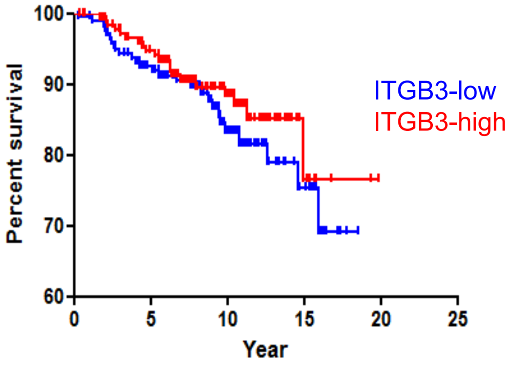

HR = 1.313 (0.7384-2.333)
p = 0.3540
Median survival, ITGB3-low (< median) = undefined
Median survival, ITGB-high (≥ median) = undefined

Disease-Free Survival (n = 815)
Median

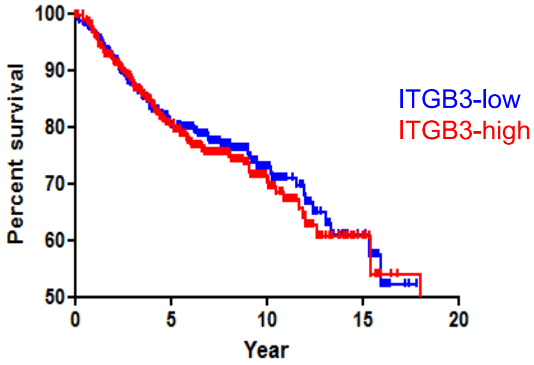

HR = 0.9344 (0.7053-1.238)
p = 0.6362
Median survival, ITGB3-low (< median) = undefined
Median survival, ITGB-high (≥ median) = 18.05 yr

Overall Survival (n = 179)
1st Quantile vs 4th Quantile

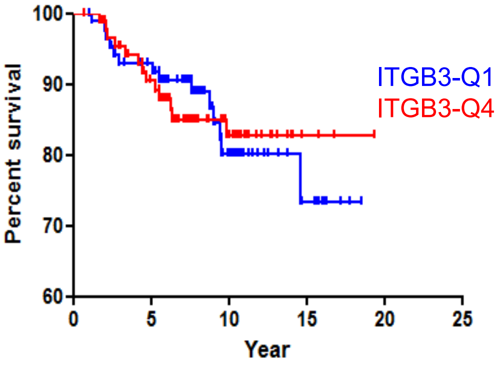

HR = 1.037 (0.4865-2.211)
p = 0.9247

Disease-Free Survival (n = 405)
1st Quantile vs 4th Quantile

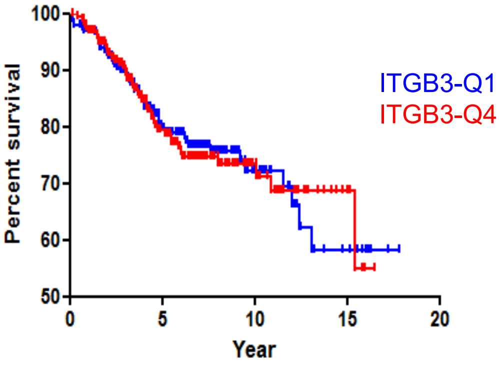

HR = 0.9857 (0.6574-1.478)
p = 0.9444

Fig. S8
