# Supplementary material for: ‘Normalizing' the malignant phenotype of luminal breast cancer cells via alpha(v)beta(3)-integrin
Source: Cell Death Dis. 2016 Dec 1;7(12):e2491–. doi: 10.1038/cddis.2016.387 (PMC5260995; doi:10.1038/cddis.2016.387)
Supplement: Supplementary Figure Legends [file cddis2016387x2.pdf]

Supplementary Figure Legends

Figure S1. Intβ3 expression in different stages of breast cancer progression. **A-B)** Paraffin fixed tissue of UDH **(A)** and IDC-G1 **(B)** stained for Int-β3 expression (red) nuclei countered stained with hematoxilin (blue). Red arrow indicates epithelial cells (luminal cells) positive for Int-β3 staining. Black arrow indicates stromal cells positive for Int-β3 staining. Bars=100 μM.

Figure S2. Characterization of MCF-7 clones and T47D cell lines for their expression of Int-αvβ3. Percentage of cells that express Int-αvβ3 (**A** & **C**) and mean fluorescence intensity (MFI) of Int-αvβ3 normalized to MCF-7 vec cells **(B)** was determined by staining the cells with PE-conjugated antibody for Int-αvβ3 and quantification was carried out with FACSdiva software for MCF-7 cell line (n=3) **(A)**, n=4 **(B)** and for T47D cell line (n=4) **(C)**; ***P $\leq$ 0.001.

Figure S3. CLPs expressing both EpCAM^{high}CD49f^{low} and Int-αvβ3 are the dominant subpopulation in MCF-7 and T47D cells. Cells derived from 2D culture of either **A-D)** MCF-7 cell lines, (**E-H)** T47D cell lines. **A & E**) Histogram representing three population of cells with different levels of expression of CD49f normalized to unstained cells. **B & F)** Mean fluorescence intensity (MFI) of CD49f expression in the different subpopulations of cells. **C & G)** Top panel: representative dot plot showing cancer stem cells expressing EpCAM^{low}CD49f^{high} (CSC), cancer luminal progenitor-like cells (CLP) either expressing EpCAM^{high}CD49f^{low} (CLP^{low}) or EpCAM^{high}CD49f^{high} (CLP^{high}) phenotype. Bottom panel (red square): representative dot plots showing CLP^{low} subpopulation positive for CD24 expression. **D) & H)** Percentage of CSC, CLP ^{low} and CLP^{high}. Quantification was carried out with FACSdiva software. Columns; mean, bars; STD; n = 3 (MCF-7 and T47D cell lines), *** P $\leq$ 0.001.

Figure S4. CSC expressing CD44^{high} CD24^{low} phenotype in MCF-7 and T47D cell lines. Cells derived from either 2D culture or from second generation of grown mammospheres of MCF-7 **(A-C)** or T47-D cell lines **(D-F). A-B & D-E)** Representative dot plots showing CSC expressing CD44^{high} CD24^{low} phenotype (red square). **C & F)** Percentage of cells expressing CD44^{high} CD24^{low} phenotype. Columns; mean, bars; STD, n = 3-7 (MCF-7 cell lines) and n=3 (T47D cell lines), * $P \leq 0.05$, ** $P \leq 0.01$ *** $P \leq 0.001$.

Figure S5. MCF-7 cell lines expressing Int-αvβ3 differentiate into acinar-like structures in the 3D BME system. A) Representative light microscopy images (magnification x20). **B)** Representative confocal images from middle section of organoids stained for F-actin (green) and nuclei (Dapi, blue). Magnification x40. Bars=50 μm. Representative results; n=10.

Figure S6. Clearing of the cells occupying the luminal space of MCF-7-Intβ3 cells is mediated partly by apoptosis A-C) CLPs^{low} derived from second generation of mammospheres generated by MCF-7 cell lines and grown in the 3D BME system. Representative confocal images of the cross-sections through the middle of organoids. Bars=50 μm. **A)** Day 19 stained with Dapi for nuclei (blue) and F-actin (green). Detached cells indicated by white arrow. **B)** Day 15 (left panel) and day 36 (right panel) stained for dead cells with EtBr (red) and for live cells with Calcein–AM (green). **C**) Left panel: scheme demonstrating the middle section of the organoids cultured in 3D system for 30 days stained for apoptotic cells (red). Right panel: organoids stained with Dapi for nuclei (blue), for F-actin (green) and for apoptotic cells with TUNEL (red) indicated by white arrow.

Figure S7. Acini developed by MCF-7-Intβ3 cells resemble UDH of the human breast tissue. A) Morphometric analysis was carried out on 4 organoids developed in the 3D BME system by

either CLP-Int-$\alpha v\beta 3^{pos}$ or CLP-Int-$\alpha v\beta 3^{neg}$. Upper panel: confocal images of the organoids stained with Dapi for nuclei (blue) were used for the analysis. Lower panel: scatter plot of the average of nuclei area analyzed. **B)** Morphometric analysis of light microscopy images (n=10; middle panel) for the roundness of the organoids described above compared to organoids developed by MCF-10A (normal breast cells) in the 3D BME system. **C-D)** Paraffin section of teratomas bearing MCF-7-vec-GFP or MCF-7-Intβ3-GFP cells subjected to H&E staining. Magnification x20, Bars=50μM.

Figure S8. Kaplan-Meier plots for the overall survival and disease-free survival of luminal-A breast cancer patients expressing Intβ3. Either median or the first and fourth quantiles were used to define low (blue; < median or first quantile) and high (red; ≥ median or fourth quantile) Intβ3 groups. Log-rank test, p-values are shown. Abbrev. HR: Hazard Ratio.
